# Use of Sex-Specific Body Mass Index to Optimize Low Correlation With Height and High Correlation With Fatness: A UK Biobank Study

**DOI:** 10.1093/aje/kwad195

**Published:** 2023-10-09

**Authors:** Qi Feng, Jean H Kim, Junqing Xie, Jelena Bešević, Megan Conroy, Wemimo Omiyale, Yushan Wu, Mark Woodward, Ben Lacey, Naomi Allen

**Keywords:** adiposity, body composition, body mass index, correlation, fatness, height, UK Biobank, weight

## Abstract

Body mass index (BMI; weight (kg)/height (m)^2^) is commonly used to measure general adiposity. However, evidence of its appropriateness for males and females remains inconsistent. We aimed to identify the most appropriate sex-specific power value that height should be raised to in the formula and the value that would make it achieve height independency and body fatness dependency. We randomly assigned UK Biobank participants recruited in the United Kingdom between 2006 and 2010 (*n* = 489,873; mean age = 56.5 years; 94.2% White) to training and testing sets (80%:20%). Using height raised to the power of −50.00 to 50.00, we identified the optimal power value that either minimized correlation with height or maximized correlation with body fat percentage, using age-adjusted correlations. The optimal power values for height were 1.77 for males and 1.39 for females. The new formulas resulted in 4.5% of females and 2.4% of males being reclassified into a different BMI category. The formulas did not show significant improvement (in terms of area under the receiver operating characteristic curve, sensitivity, and specificity) in identifying individuals with excessive body fat percentage or in predicting risk of all-cause mortality. Therefore, the conventional BMI formula is still valuable in research and disease screening for both sexes.

## Abbreviations

BF%: body fat percentage
BIA: bioimpedance analysis
BMI: body mass index
CI: confidence interval
DXA: dual-energy x-ray absorptiometry
FFM: fat-free mass
FM: fat mass
SD: standard deviation
WC: waist circumference
WHR: waist:hip ratio
WHtR: waist:height ratio

Obesity has long been established as causally related to a wide range of adverse health outcomes, such as cardiovascular diseases (1), diabetes (2), cancers (3), infections (4), and overall mortality (5). Excessive body fat, rather than actual body weight, is considered the primary factor associated with poorer health; central obesity measures, including waist circumference (WC), waist:hip ratio (WHR), and waist:height ratio (WHtR), have also been found to be independent predictors of various health outcomes (6, 7). There exist various indices that attempt to determine levels of fatness (fatness dependency) while removing the effect of height (height independency) in classifying individuals as overweight or obese (8).

The most widely used measure of weight relative to height is body mass index (BMI). Introduced by Adolphe Quetelet in 1832 as a formula for determining expected weight from height during human growth, BMI is calculated as weight in kilograms divided by squared height in meters (9). The name was coined by Ancel Keys in 1972, when he found the formula to be an appropriate index of body fatness in males (8). In calculating relative weight, height independency has been regarded as more important than fatness dependency (10). Keys found that compared with weight/height and weight/height^3^, the index with height raised to a power of 2.00 was least correlated with height while maintaining high correlation with skinfold thickness (8). However, that analysis only considered those 3 candidate power values and thereby missed many other possibilities, such as other integers, fractions, or negative numbers. Furthermore, the study by Keys et al. included only male participants (8), which may have limited generalizability to females.

It is unclear whether the optimal power of height is the same for males and females, considering the sexual dimorphism in fat accumulation and distribution. For the same BMI level, females typically have more body fat, especially subcutaneous adipose tissue in the gluteofemoral area, whereas males tend to have more visceral adipose tissue around the abdomen and waist (11–13). Little is known about how such sex-specific differences in body fat accumulation and distribution might affect the calculation of BMI. Furthermore, little research has been performed as to whether there are more suitable values for the power of height to achieve height independency and fatness dependency. Although some studies have touched on these questions, research gaps remain. For example, Benn et al. (10) calculated the most suitable value for the power of height (1.8) but included only male participants. Florey et al. (14) included both male and female participants but only compared 3 candidate values for power (1, 2, and 3) and found that the optimal power was 2 for males and 1 for females. Other studies similarly found that the optimal power value was lower for females than for males (15, 16). For body fatness measures, previous studies have commonly used body weight (15), skinfold thickness (16), and densitometry-derived body fat mass (17); however, novel techniques such as bioimpedance analysis (BIA) and dual-energy x-ray absorptiometry (DXA) can measure body composition more accurately and easily (18). Furthermore, few previous studies have considered the correlations of alternative BMI formulas with central obesity measures.

In this study, we aimed to explore the optimal sex-specific power value for height in the BMI formula for meeting height-independency and fatness-dependency criteria in a contemporary European population, using highly accurately measured body composition.

## METHODS

This analysis involved identifying sex-specific optimal power values that height was raised to in the calculation of BMI in a training set. If sex-specific optimal power values were different from 2.00, we investigated how the new formula(s) would change the accuracy of identifying individuals with excessive body fat and how well the new formula(s) would work for risk prediction for all-cause mortality in testing sets.

### Settings

The UK Biobank is a prospective cohort study of half a million participants from England, Scotland, and Wales who were aged 40–69 years at recruitment between 2006 and 2010 (19). All participants provided written informed consent. Information on socioeconomic status, lifestyle, and medical history was collected through touch-screen questionnaires and verbal interviews. Participants also provided biological samples and underwent a number of physical measurements, including weight, height, and BIA. Further, up to 100,000 participants are currently undertaking an imaging assessment (2014–2024), which includes a full-body DXA scan that provides detailed measures of body composition.

### Measurement of body composition

Body composition measures included fat mass (FM), fat-free mass (FFM), and body fat percentage (BF%; calculated as FM/body weight). These measures were assessed using BIA for all UK Biobank participants at recruitment (2006–2010) and using DXA for about 35,000 participants who undertook an imaging assessment (2014–2022).

BIA was performed with the Tanita BC-418 MA Body Composition Analyzer (Tanita Corporation, Tokyo, Japan) (20). The analyzer measured body impedance with a high-frequency current (50 kHz) and 8 contact electrodes. Participants were asked to place their bare feet on the analyzer platform, to keep their feet still and in good contact with the platform’s metallic electrodes, and to use their hands to grip the two handles firmly, with palm and thumb contacting the metallic electrodes and arms hanging loosely by their sides. Weight and body composition data were automatically generated by the analyzer.

DXA was performed with the Lunar iDXA Scanner (GE Healthcare, Milwaukee, Wisconsin) during an imaging assessment performed some years after recruitment into the study (from 2014 onwards) (21). Participants were asked to lie flat on their backs on the scanning couch for a whole-body scan. All measurement was performed by trained radiographers according to standard procedures.

WC and hip circumference were measured with a Seca 200-cm tape measure (Seca GmbH & Co. KG, Hamburg, Germany), where participants were asked to stand with feet facing directly forward and shoulder-width and with arms folded across the chest. WC was measured on an out-breath using the tape measure around the participant’s waist, just above the hip bones. Hip circumference was measured at the widest part of the hips. Standing height was measured using a 240-cm telescopic height rod device (Seca 202; Girod Medical, Carquefou, France), and participants were asked to stand barefoot with their backs against the vertical scale, feet parallel to each other, toes pointing forward and soles flat on the floor. The posture was checked to ensure that the participant was standing upright and unsupported, with legs straight and arms by the sides, and with buttocks and shoulder blades touching the vertical scale. WHR was calculated as WC divided by hip circumference. WHtR was calculated as WC divided by standing height.

### Measurement of other variables

Ethnicity was categorized into White, Asian, Black, and other. Socioeconomic status was measured using educational attainment (less than secondary school, secondary school, vocational school, or higher education) and the Townsend deprivation index (a postcode-derived measure of socioeconomic status). Tobacco smoking and alcohol drinking status were self-reported as previous user, current user, or never user.

### Statistical analysis

We included all participants who 1) were not pregnant, 2) had not withdrawn from the UK Biobank study, and 3) had no missing data for BIA-measured body composition. We randomly assigned 80% of the eligible participants to the training set and the remaining 20% to a testing set, stratified by sex. Participants with DXA-measured body composition data were used as a second testing set, since DXA is considered more accurate than BIA in measuring body composition (22) (see Web Figure 1, available at https://doi.org/10.1093/aje/kwad195).

In the training set, we aimed to find the optimal power value for height in the BMI formula that was needed to achieve height independency and fatness dependency.

$$ \mathrm{BMI}=\frac{\mathrm{weight}\ }{{\mathrm{height}}^{\mathrm{power}}}. $$

We changed the power value from −50.00 to 50.00 in increments of 0.01 and calculated age-adjusted partial Pearson correlation coefficients and 95% confidence intervals (CIs) for correlations with height, body weight, body composition measures (FM, FFM, and BF%), and central adiposity measures (WC, WHR, and WHtR). BF% was the primary measure of fatness. Optimal power values were identified separately on the basis of various criteria: height independency (i.e., when the correlation with height was 0) or fatness dependency (i.e., when the correlation with a body fatness measure was maximized). Among all of the power values identified under these criteria, we selected one that had the most appropriate properties of a low correlation with height and a high correlation with fatness measures. We performed correlation analysis in males and females separately to identify sex-specific optimal power values. Because the optimal power value was different from 2, we derived new sex-specific BMI formulas.

We evaluated the correlations of the old and new formulas with anthropometric measures in the main testing set (comprising 20% of the study population), overall and by ethnic group, as well as in the testing set of eligible individuals with DXA-measured body composition.

We evaluated the accuracy of the old and new BMI values in identifying individuals with excessive body fat (23, 24), defined as BF% > 35% for females and BF% > 25% for males—measures that are widely used in health research and clinical practice (25). We first estimated the area under the receiver operating characteristic curve for each of the formulas, by fitting a logistic regression of the old and new BMI values on BF% levels (high vs. low). We then identified optimal cutoffs that maximized the Youden index value (the sum of sensitivity and specificity). We stratified the analysis by relative sitting height, calculated as sitting height divided by standing height, since previous evidence suggested that relative sitting height might be a potential effect modifier in the relationship between BMI and BF% (26–28). Both sensitivity and specificity are important in assessing screening tools, but we considered sensitivity to be more important than specificity, because missing an individual with high BF% may lead to delay in health intervention and potentially worse outcomes, whereas individuals who are falsely classified as obese can still benefit from early warning and lifestyle intervention to improve health (24).

Furthermore, we evaluated the associations of all-cause mortality with the old and new BMI formulas to see whether the new formula could improve risk prediction. In the UK Biobank study, participants were followed up via linkage to the UK national death registry. The censoring date was defined as the date of death or the last date of follow-up (November 30, 2022), whichever occurred earlier. A Cox proportional hazards model was fitted to estimate hazard ratios and 95% CIs, adjusted for age, Townsend deprivation index, education, smoking, alcohol drinking, and ethnicity. Conventional and sex-specific BMI values were categorized into fifths, and the lowest fifth was used as the reference category. Model fit was measured using the Akaike information criterion, with a lower value indicating a better model fit.

All analyses were performed in R, version 3.6.0 (R Foundation for Statistical Computing, Vienna, Austria).

### Ethics approval

Research Tissue Bank approval was obtained from the governing Research Ethics Committee for the UK Biobank study, as recommended by the National Research Ethics Service. Permission to use the UK Biobank Resource was approved by the access subcommittee of the UK Biobank Board.

## RESULTS

Among 489,873 eligible participants (mean age = 56.5 years; 54.6% female), males had a higher WC (mean = 97.0 (standard deviation (SD), 11.2) cm vs. 84.7 (SD, 12.5) cm), higher FFM (63.7 (SD, 7.8) kg vs. 44.5 (SD, 5.0) kg), and higher BMI (27.9 (SD, 4.2) vs. 27.1 (SD, 5.2)) than females but lower FM (22.3 (SD, 8.2) kg vs. 27.0 (SD, 10.1) kg). These characteristics were similar between the training set (213,901 females and 177,996 males) and the main testing set (53,476 females and 44,500 males) (Table 1). The second testing set, which was comprised of participants with DXA measurements (*n* = 35,390), had a higher BF% than the overall cohort (39.0% (SD, 7.3) vs. 36.6% (SD, 6.9) for females; 31.0% (SD, 6.6) vs. 25.3% (SD, 5.7) for males) (Web Table 1). Conventional BMI was highly correlated with FM, BF%, WC, and WHtR, and moderately correlated with FFM and WHR, across both sexes and all age subgroups (Web Table 2). A stronger inverse correlation of BMI with height was seen in females than in males (−0.13 vs. –0.06).

**Table 1:** Characteristics of Study Participants From the UK Biobank Included in an Analysis of Sex-Specific Body Mass Index, United Kingdom, 2006–2010

	**Sex and Participant Subgroup**														
	**Female**						**Male**								
	**Training Set** **(*n* = 213,901)**			**Testing Set** **(*n* = 53,476)**			**Training Set** **(*n* = 177,996)**			**Testing Set** **(*n* = 44,500)**			**Total** **(*n* = 489,873)**		
**Variable**	**Mean (SD)**	**No.**	**%**	**Mean (SD)**	**No.**	**%**	**Mean (SD)**	**No.**	**%**	**Mean (SD)**	**No.**	**%**	**Mean (SD)**	**No.**	**%**
Age, years	56.3 (8.0)			56.4 (8.0)			56.7 (8.2)			56.7 (8.2)			56.5 (8.1)		
Townsend deprivation index^a^	1.35 (3.0)			−1.33 (3.0)			−1.28 (3.1)			−1.26 (3.1)			−1.31 (3.1)		
Height, cm	162 (6.3)			162 (6.3)			176 (6.8)			176 (6.8)			168 (9.3)		
Hip circumference, cm	103 (10.3)			103 (10.3)			103 (7.5)			103 (7.5)			103 (9.2)		
Waist circumference, cm	84.7 (12.5)			84.7 (12.5)			97.0 (11.2)			97.0 (11.2)			90.3 (13.4)		
Weight, kg^b^	71.4 (14.0)			71.5 (14.0)			86.0 (14.2)			86.0 (14.2)			78.1 (15.9)		
Fat mass, kg^b^	26.9 (10.1)			27.0 (10.0)			22.3 (8.2)			22.3 (8.2)			24.9 (9.5)		
Fat-free mass, kg^b^	44.5 (5.0)			44.5 (5.0)			63.7 (7.8)			63.7 (7.8)			53.2 (11.5)		
Body fat percentage, %	36.6 (6.9)			36.6 (6.9)			25.3 (5.7)			25.3 (5.7)			31.5 (8.5)		
Ethnicity															
White		201,731	94.3		50,447	94.3		167,621	94.2		41,878	94.1		461,677	94.2
Asian		4,360	2.0		1,042	1.9		4,434	2.5		1,119	2.5		10,955	2.2
Black		3,546	1.7		908	1.7		2,654	1.5		621	1.4		7,729	1.6
Other		3,433	1.6		864	1.6		2,337	1.3		598	1.3		7,232	1.5
Unknown		831	0.4		215	0.4		950	0.5		284	0.6		2,280	0.5
Alcohol drinking															
Current drinker		193,259	90.3		48,459	90.6		166,585	93.6		41,563	93.4		449,866	91.8
Never drinker		12,425	5.8		2,998	5.6		4,840	2.7		1,234	2.8		21,497	4.4
Previous drinker		7,710	3.6		1,905	3.6		6,120	3.4		1,590	3.6		17,325	3.5
Unknown		507	0.2		114	0.2		451	0.3		113	0.3		1,185	0.2
Tobacco smoking															
Current smoker		19,060	8.9		4,703	8.8		21,892	12.3		5,636	12.7		51,291	10.5
Never smoker		126,939	59.3		31,684	59.2		87,001	48.9		21,568	48.5		267,192	54.5
Previous smoker		66,880	31.3		16,825	31.5		68,209	38.3		17,033	38.3		168,947	34.5
Unknown		1,022	0.5		264	0.5		894	0.5		263	0.6		2,443	0.5
Education															
Less than secondary school		35,842	16.8		8,930	16.7		30,186	17.0		7,627	17.1		82,585	16.9
Secondary school		41,313	19.3		10,203	19.1		23,974	13.5		5,927	13.3		81,417	16.6
Vocational school		12,341	5.8		3,117	5.8		8,930	5.0		2,238	5.0		26,626	5.4
Higher education		54,284	25.4		13,668	25.6		51,664	29.0		12,907	29.0		132,523	27.1
Unknown		66,094	30.9		16,590	31.0		59,739	33.6		14,941	33.6		157,364	32.1

Abbreviation: SD, standard deviation.

^a^ The Townsend deprivation index is a postcode-based measure of socioeconomic status, with a smaller value indicating a lower level of deprivation.

^b^ Weight, fat mass, and fat-free mass were measured with bioimpedance analysis.

**Figure 1:**
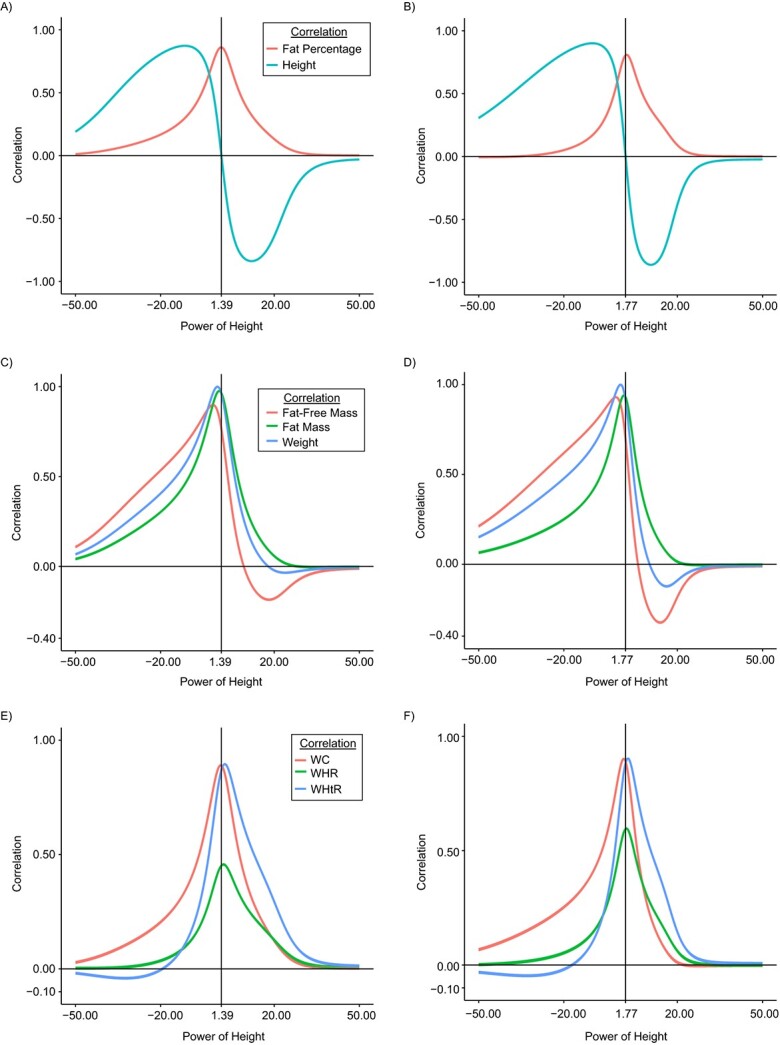
Age-adjusted correlations between relative weight indices and height and obesity measures among females (left) and males (right) in the UK Biobank, United Kingdom, 2006–2010. Top row: correlations with fat mass percentage and height in females (A) and males (B). Middle row: correlations with fat-free mass, fat mass, and weight in females (C) and males (D). Bottom row: correlations with waist circumference (WC), waist:hip ratio (WHR), and waist:height ratio (WHtR) in females (E) and males (F). The graphs show age-adjusted correlation coefficients and their 95% confidence intervals. The vertical lines indicate the optimal power values that make the correlation with height 0. Fat mass and fat-free mass were measured via bioimpedance analysis.

When power for height in the calculation of BMI changed from −50.00 to 50.00, the correlation with height and fatness measures changed substantially (Figure 1). The correlation curve for height showed a wave-shaped pattern (upward-downward-upward) and crossed the reference line *y* = 0 once only, making it possible to find an optimal power for the height-independency criterion. The correlation curves for body fatness measures showed an approximately bell-shaped pattern for BF%, FM, WC, WHR, and WHtR but a wave-shaped (upward-downward-upward) pattern for weight and FFM.

For females, to achieve 0 correlation with height, the optimal power value was calculated to be 1.39. The optimal power values for achieving maximal correlation with the various adiposity measures ranged greatly, from −1.58 to 2.60, and the BMI values resulting from these power values showed substantial variation in the correlations with height; correlation coefficients ranged between −0.24 and 0.51 (Figure 1, Table 2). As compared with the height-independent BMI (power = 1.39), the other power values generally yielded BMI values that had similar or marginally improved correlation with adiposity measures. However, raising height to a power of −1.58, to maximize correlation with FFM, produced substantially lower correlations with BF%, FM, WC, WHR, and WHtR while greatly increasing the correlation with height. By contrast, raising height by a power of 1.33 (maximizing the correlation with BF%) or 1.16 (maximizing the correlation with WC) yielded nearly identical correlations with the height-independent BMI but higher correlation with height. Hence, raising height to a power of 1.39 demonstrated improved performance over the other power values, since there were marginal differences in correlations with the various adiposity measures while maintaining height independence, which gave the optimal power formula—denoted here as BMI_new_female—as weight/height^1.39^.

**Table 2:** Age-Adjusted Correlation Coefficients for the Correlation of Body Mass Index With Measures of Height and Body Fatness, Using Different Power Values for Height in Calculating Body Mass Index, UK Biobank, United Kingdom, 2006–2010

		**Correlation Coefficient**							
**Sex and Minimization or Maximization Criterion** ^**a**^	**Power to Which Height Was Raised**	**Height**	**BF%**	**Weight**	**FM**	**FFM**	**WC**	**WHR**	**WHtR**
Female									
Old BMI formula^b^	2.00	−0.12	0.85	0.92	0.94	0.70	0.88	0.46	0.89
Height	1.39	0.00	0.86	0.96	0.96	0.77	0.89	0.45	0.87
BF%	1.33	0.01	0.86	0.96	0.97	0.77	0.89	0.45	0.87
Weight	−0.01	0.27	0.83	1.00	0.97	0.87	0.87	0.41	0.78
FM	0.63	0.15	0.85	0.99	0.98	0.83	0.89	0.44	0.82
FFM	−1.58	0.51	0.74	0.96	0.90	0.90	0.78	0.35	0.63
WC	1.16	0.05	0.86	0.97	0.97	0.79	0.89	0.45	0.86
WHR	2.14	−0.15	0.85	0.91	0.93	0.69	0.87	0.46	0.89
WHtR	2.60	−0.24	0.83	0.87	0.90	0.63	0.85	0.45	0.90
Male									
Old BMI formula^b^	2.00	−0.06	0.81	0.88	0.92	0.66	0.88	0.60	0.89
Height	1.77	0.00	0.80	0.91	0.93	0.69	0.89	0.59	0.88
BF%	2.16	−0.10	0.81	0.86	0.91	0.63	0.87	0.60	0.90
Weight	−0.01	0.41	0.70	1.00	0.90	0.89	0.87	0.52	0.73
FM	1.18	0.15	0.78	0.96	0.94	0.78	0.90	0.58	0.84
FFM	−1.65	0.65	0.55	0.96	0.79	0.93	0.76	0.42	0.54
WC	1.13	0.16	0.78	0.97	0.94	0.79	0.90	0.58	0.84
WHR	2.07	−0.08	0.81	0.88	0.92	0.65	0.88	0.60	0.89
WHtR	2.60	−0.21	0.80	0.80	0.88	0.55	0.84	0.59	0.90

Abbreviations: BF%, body fat percentage; BMI, body mass index; FFM, fat-free mass; FM, fat mass; WC, waist circumference; WHR, waist:hip ratio; WHtR, waist:height ratio.

^a^ The table shows the optimal power values identified under different criteria and the age-adjusted correlations of various BMI formulas with height and body fatness measures. For the height criterion, the goal was to find the power value that minimized the correlation with height. For the criteria of body fatness measures, the goal was to find the power value that maximized the correlation with the fatness measure. The 95% confidence intervals for the correlation coefficients are not shown here, as they all were very narrow.

^b^ Weight (kg)/height (m)^2^.

For males, the optimal power value needed to achieve height independency was 1.77. The optimal power values for achieving maximal correlation with the various adiposity measures ranged from −1.65 to 2.60, and the according BMI values resulting from these power values showed substantial variation in the correlations with height, with correlation coefficients ranging between −0.21 and 0.65 (Figure 1, Table 2). As compared with the optimal power needed to achieve height independency (power = 1.77), the other power values yielded BMI values that had similar or marginally improved correlation with adiposity measures. However, raising height to a power of −1.65 (to maximize correlation with FFM) showed substantially lower correlations with BF%, FM, WC, WHR, and WHtR while greatly increasing the correlation with height. By contrast, a power of 2.16 (maximizing correlation with BF%) or 2.07 (maximizing correlation with WHR) yielded similar correlations with the height-independent BMI but higher correlation with height. Hence, the most optimal power formula—denoted here as BMI_new_male—was weight/height^1.77^.

**Figure 2:**
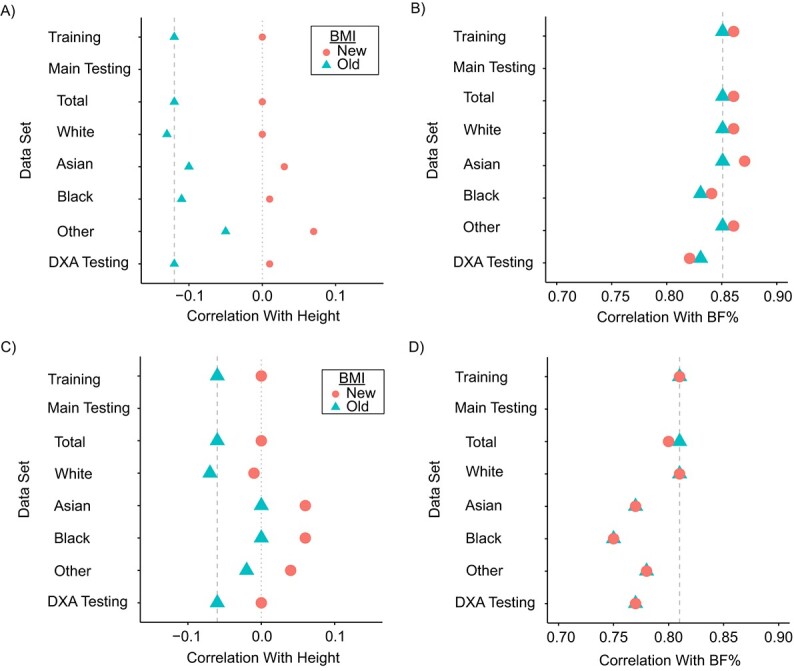
Age-adjusted correlation coefficients for the correlation of old and new body mass index (BMI) formulas with height and body fat percentage (BF%), by sex and ethnicity, in the UK Biobank, United Kingdom, 2006–2022. A) Correlation with height in females; B) correlation with BF% in females; C) correlation with height in males; D) correlation with BF% in males. “Old” BMI represents the conventional BMI formula, weight (kg)/height (m)^2^. “New” BMI represents a new BMI formula derived on the basis of height-independency criteria—weight (kg)/height (m)^1.39^ for females and weight (kg)/height (m)^1.77^ for males. Ethnicity-specific testing sets were subsets of the main testing set, in which body composition was measured with bioimpedance analysis. In the DXA testing set, body composition was measured with dual-energy x-ray absorptiometry (DXA).

The correlations of the new and old BMI values with height and fatness measures in the testing sets are shown in Figure 2 (and Web Table 3). The correlations of the BMI formulas with height were virtually identical between the training set and both testing sets. However, while the correlations of BMI values with BF% were similar between the training set and the main testing set, the DXA testing set showed lower correlations (0.82 vs. 0.86 for females and 0.77 vs. 0.81 for males, respectively).

Across all ethnic groups, BMI_new demonstrated a low correlation with height and a high correlation with BF%, although small variations existed. For example, while BMI_new showed greater height independency for females of all ethnicities, for males the new formula did not demonstrate superior height independency in Black and Asian ethnic groups. Compared with White males, all other ethnic groups showed higher correlation of BMI_new with height and lower correlation with BF% (Figure 2). BMI_new showed higher correlation with height, particularly in the “other” ethnic group (0.07), as compared with White females (0.00), while correlations with BF% were slightly higher among Asian females (0.87) and lower among Black females (0.84).

The BMI_new mean values were 24.0 (SD, 1.1), 30.6 (SD, 2.2), 36.5 (SD, 2.0), and 46.1 (SD, 5.6) for conventional BMI-defined underweight, normal-weight, overweight, and obese categories, respectively, in females and 20.3 (SD, 0.7), 26.4 (SD, 1.6), 31.1 (SD, 1.6), and 37.9 (SD, 3.8), respectively, in males (Web Figure 2).

In the main training set, 60.0% of females and 52.9% of males had excessive BF%. BMI_old and BMI_new yielded similar areas under the receiver operating characteristic curve for BF% in both females (0.93 vs. 0.94) and males (0.95 vs. 0.95), which was consistent across relative sitting height subgroups (Table 3). For females, under the optimal cutoff values for maximizing the Youden index (34.2 for BMI_new, 25.4 for BMI_old), BMI_new yielded marginally higher specificity (86.3% vs. 85.3%) but similar sensitivity (84.8% vs. 84.9%). For males, using the optimal cutoff values (31.1 for BMI_new, 27.4 for BMI_old), BMI_new yielded similar specificity (91.6% vs. 91.7%) but slightly higher sensitivity (86.9% vs. 85.8%). Using BMI_new formulas and cutoff values resulted in 4.5% of females and 2.4% of males becoming reclassified into a different BMI category. More specifically, 2.4% of females were reclassified into a lower BMI category and 2.1% were reclassified into a higher BMI category; for males, the reclassifications were 0.7% and 1.7%, respectively.

**Table 3:** Accuracy of Old and New Body Mass Index Formulas in Identifying Individuals With a High Body Fat Percentage^a^ in a Testing Data Set From the UK Biobank, United Kingdom, 2006–2010

	**No. of** **Persons**	**AUC**		**Sensitivity, %**		**Specificity, %**	
**Sex and RSH** ^**b**^ **Group** ^**c**^		**Old BMI** ^**d**^	**New BMI** ^**e**^	**Old BMI**	**New BMI**	**Old BMI**	**New BMI**
Female	53,476	0.93	0.94	84.9	84.8	85.3	86.3
Lower third (<0.50)	1,305	0.91	0.92	88.3	84.8	75.6	81.5
Middle third (0.50–0.55)	50,429	0.93	0.94	84.9	84.8	85.6	86.5
Upper third 3 (≥0.56)	1,336	0.94	0.95	85.7	88.4	89.7	88.2
Male	44,500	0.95	0.95	85.8	86.9	91.7	91.6
Lower third (<0.50)	1,169	0.94	0.95	88.4	90.7	91.0	89.6
Middle third (0.50–0.54)	41,181	0.95	0.96	85.6	86.8	92.0	91.8
Upper third (≥0.55)	1,807	0.94	0.95	91.1	92.1	87.8	85.7

Abbreviations: AUC, area under the receiver operating characteristic curve; BMI, body mass index; RSH, relative sitting height.

^a^ High body fat percentage was defined as having a body fat percentage > 35% in females and > 25% in males.

^b^ RSH, defined as sitting height divided by standing height, is a measure of body build.

^c^ The cutoff values for categorizing RSH into thirds were the 2.5% and 97.5% quantiles.

^d^ Conventional BMI formula (weight (kg)/height (m)^2^).

^e^ New BMI formula derived on the basis of height-independency criteria—weight (kg)/height (m)^1.39^ for females and weight (kg)/height (m)^1.77^ for males.

**Figure 3:**
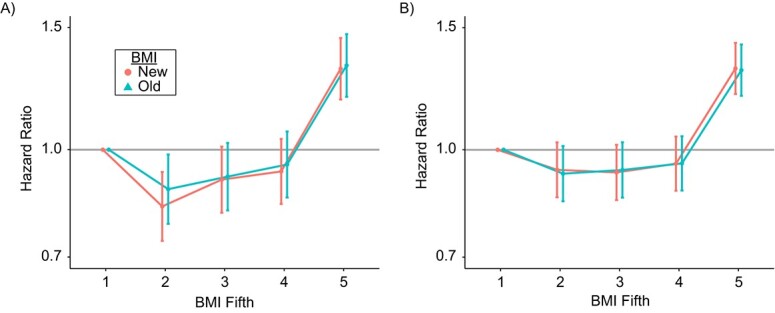
Hazard ratios for associations between all-cause mortality and new- and old-formula–derived body mass index (BMI) among females (A) and males (B) in the UK Biobank, United Kingdom, 2006–2022. “Old” BMI represents the conventional BMI formula, weight (kg)/height (m)^2^. “New” BMI represents a new BMI formula derived on the basis of height-independency criteria—weight (kg)/height (m)^1.39^ for females and weight (kg)/height (m)^1.77^ for males. The Cox model used to estimate hazard ratios adjusted for age, Townsend deprivation index, education, tobacco smoking, alcohol drinking, and ethnicity. Akaike information criterion values for the old and new BMI formulas and were 73,871 and 73,862, respectively, in females and 103,577 and 103,586, respectively, in males. Error bars show 95% confidence intervals.

We compared the association and risk prediction of BMI_old and BMI_new for all-cause mortality. During a median follow-up period of 12.0 years, 3,530 females and 5,043 males died. Both BMI formulas demonstrated similar hazard ratio estimates and model fits. For females, the hazard ratios for the highest fifth of BMI versus the lowest were 1.32 (95% CI: 1.19, 1.47) for BMI_old and 1.31 (CI: 1.18, 1.45) for BMI_new. Among males, the corresponding hazard ratios were 1.30 (95% CI: 1.20, 1.42) and 1.31 (95% CI: 1.20, 1.43), respectively (Figure 3, Web Table 4).

## DISCUSSION

Precise quantification of overweight and obesity status is crucial for identifying high-risk populations to be targeted for preventative health measures. BMI (weight/height^2.00^) has been widely used at both the individual and population levels to assess adiposity because of its easy calculation and strong correlation with body fatness measures. Previous investigators have commonly used allometric analysis to investigate how body weight scales to height and thus decide on a power value via regression models (10, 15, 16, 29). However, allometric models rely on the assumption that standard weight is scaled to height. By contrast, using bioimpedance measures in a large-scale general population cohort and basing analyses on the criteria of height independency and fatness dependency, we calculated the optimal formulas to be weight/height^1.39^ for females and weight/height^1.77^ for males. These two approaches generated consistent findings that the optimal power value was lower in females than in males, and that the power value in males is closer to 2 (15, 16, 30). However, these new formulas showed marginal differences regarding their correlation with body fatness measures, accuracy in identifying individuals with high BF%, and estimation of body fat’s association with all-cause mortality.

Our study used correlation curves to visualize the relationship between power for height and the correlation of BMI with height and fatness measures. These correlation curves lend strong support for prioritizing the height-independency criterion over fatness dependency. It was noted that the correlation curves yielded only 1 optimal power value that minimized the correlation with height (i.e., a single intercept of the correlation curve of height with *y =* 0), whereby the correlations with fatness measures were close to their peaks. Moreover, where correlation_height_ = 0, the correlation curve for height decreased more rapidly (i.e., larger slope) than the curves for fatness measures, reinforcing the rationale for prioritizing height independency over fatness dependency. When done so, correlation_FM_ remains high (0.93 and 0.96 for males and females, respectively). Our findings therefore provide robust evidence for the rationale to generate a BMI index that is independent of height while being correlated with body fatness (10).

We found that the optimal power for a BMI formula difference between the sexes was 1.39 in females and 1.77 (i.e., closer to the existing formula of 2) in males, which is consistent with previous work (14–16). A possible reason for this difference may include the sexual dimorphism in fat accumulation and distribution. Historically, the justification for using the power of 2 in the original BMI formula was derived mainly from data collected among males, while its external validity in females has rarely been evaluated, despite BMI’s widespread use. Our findings helped to address the underrepresentation of females in the derivation of the BMI formula and showed that the optimal power value was lower for females than for males.

Compared with the original BMI formula, the new BMI formulas are more independent of height, while maintaining similarly high correlations with body fatness measures. This finding was largely consistent across different ethnic groups, with the exception of Asian and Black males, in whom the original BMI formula was slightly more independent of height; however, it seems unlikely that this small difference would change risk prediction for all-cause mortality, although future studies with larger sample sizes are warranted to investigate other health outcomes.

We found no evidence that the new BMI formulas significantly improved the sensitivity and specificity of identifying individuals with high BF%, suggesting that it would not outperform the original formula if applied in a clinical setting to screen for obesity. Further, the new BMI formulas reclassified less than 5% of the population into a different BMI category, leading to minimal change in the association of categorical BMI with all-cause mortality. However, it remains uncertain whether the new BMI formulas would improve risk prediction for other health outcomes; this question warrants additional research.

Given the easy and widespread use of the conventional BMI formula in epidemiologic research, health screening, and disease management, we recommend that future research continue using the conventional BMI formula, weight/height^2^, for both sexes. To address its limitation of being unable to distinguish body composition and body shape, which has been widely recognized, additional measures can be used together with BMI, since measurement of central obesity and body composition has become much easier in practice. For example, UK weight management guidelines have recommended combining BMI and WHtR (31).

We acknowledge some limitations in this study. The generalizability of our findings may be limited by the fact that the UK Biobank cohort is a middle-aged, primarily White cohort with higher socioeconomic status and better health status than the general population (32). Therefore, caution should be taken when generalizing these findings to populations with different characteristics. Although we performed subgroup analysis based on ethnicity, the sample sizes for Asian and Black females and males were small. Future studies with bigger sample sizes are required to investigate the potential ethnic differences. We examined potential power values in a range of −50 to 50, and this range was arbitrary; however, the resulting correlation curves showed the patterns clearly and indicated that expanding beyond these limits is unlikely to change the substantive findings.

In conclusion, we derived new BMI formulas to optimize low correlation with body height and high correlation with fatness. However, in comparison with the old BMI formula, there is currently no evidence that the new formulas showed substantial improvement regarding correlation with fatness measures, identifying individuals with high BF%, and predicting risk of all-cause mortality. Given the easy and widespread use of the old BMI formula, it remains a valuable screening tool for obesity in research and practice.

## Supplementary Material

Web_Material_kwad195
