# Supplementary material for: Use of Sex-Specific Body Mass Index to Optimize Low Correlation With Height and High Correlation With Fatness: A UK Biobank Study
Source: Am J Epidemiol. 2023 Oct 9;193(2):296–307. doi: 10.1093/aje/kwad195 (PMC10840076; doi:10.1093/aje/kwad195)
Supplement: Web_Material_kwad195 [file web_material_kwad195.zip › kwad195 Feng Web Material.pdf]

Web Material

Use of Sex-Specific Body Mass Index to Optimize Low Correlation With Height and High Correlation With Fatness: A UK Biobank Study

Qi Feng, Jean H. Kim, Junqing Xie, Jelena Bešević, Megan Conroy, Wemimo Omiyale, Yushan Wu, Mark Woodward, Ben Lacey, and Naomi Allen

Contents

Web Figure 1: Flowchart for participant selection 2

Web Table 1: Summary statistics of the study participants with DXA measurement for testing set. 3

Web Table 2: Age-adjusted correlation between conventional body mass index and height, fat mass, fat free mass, waist circumference and fat mass percentage 4

Web Table 3: Age-adjusted correlation coefficients of old and new BMI with height and fatness measures in females and males in training and testing data sets 5

Web Figure 2: Box plot of conventional and female-specific BMI according to weight groups defined by conventional BMI in females 7

Web Table 4: Association between all-cause death with old and new BMI in females and males in the testing set 8

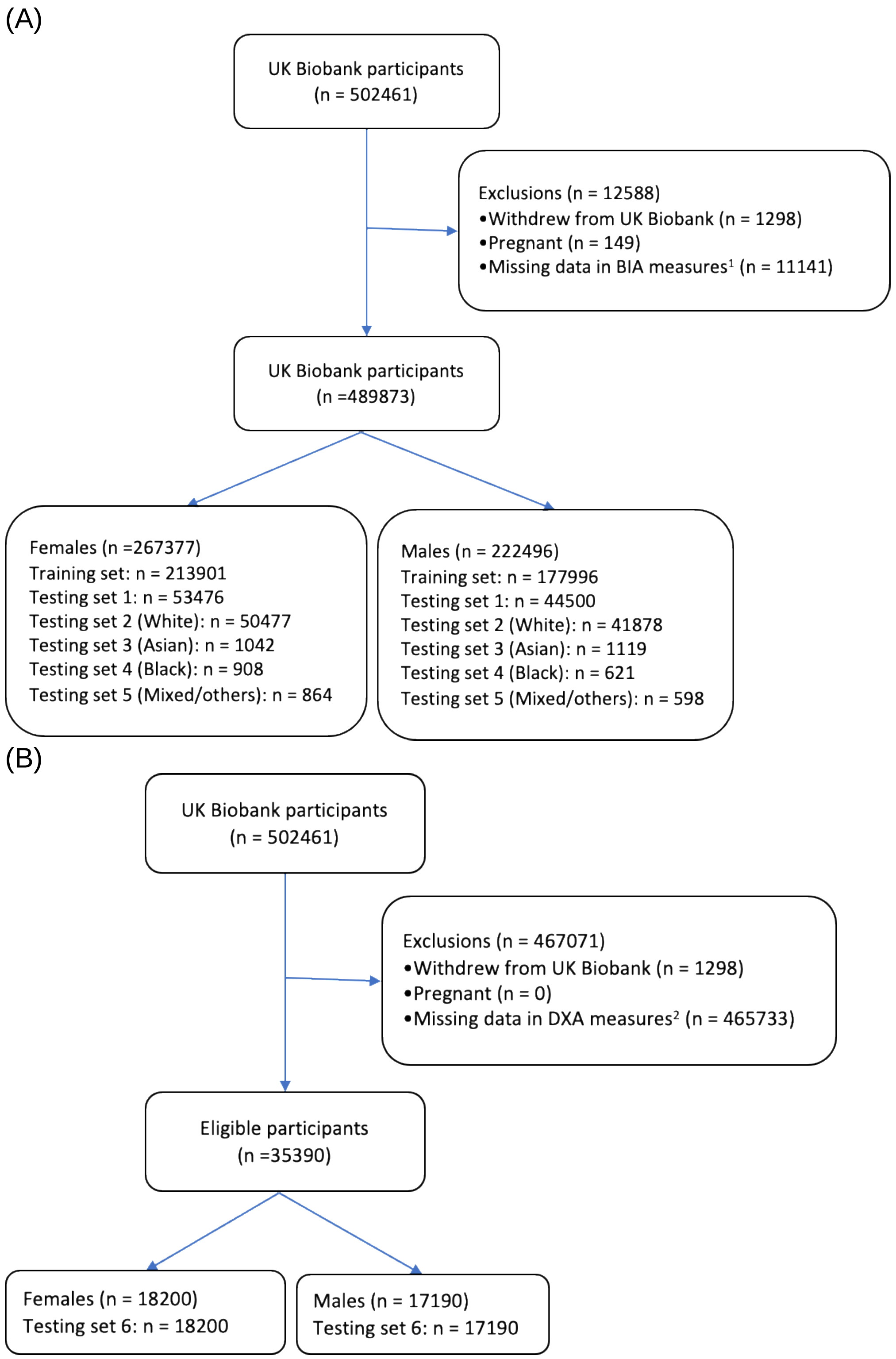

Web Figure 1: Flowchart for participant selection

(A) participant selection for the training set and the main testing set. (B) participant selection for the DXA testing set. 1: BIA measured fat mass, fat free mass, body weight, and body fat percentage. 2: DXA measured fat mass, fat free mass and body fat percentage. BIA: bioimpedance analysis. DXA: dual-energy X-ray absorptiometry.

Web Table 1: Summary statistics of the study participants with DXA measurement for testing set

	Females (*n* = 18200)	Males (*n* = 17190)	Overall (*n* = 35390)
Age (years)	54.5 (7.5)	56.0 (7.7)	55.2 (7.6)
Townsend Deprivation Index	-1.83 (2.7)	-1.93 (2.7)	-1.88 (2.7)
Height (cm)	163 (6.28)	176 (6.65)	169 (9.28)
Hip circumference (cm)	101 (9.9)	100 (7.4)	100 (8.8)
Waist circumference (cm)	82.1 (11.8)	93.8 (10.8)	87.8 (12.7)
Weight (kg)	69.1 (13.2)	83.8 (13.6)	76.2 (15.3)
Fat mass (kg)	26.5 (9.6)	24.7 (8.9)	25.6 (9.3)
Fat free mass (kg)	41.9 (4.9)	58.4 (6.8)	49.9 (10.2)
Body fat percent (%)	39.0 (7.3)	30.1 (6.6)	34.7 (8.3)
Ethnicity			
White	17533 (96.3%)	16540 (96.2%)	34073 (96.3%)
Asian	237 (1.3%)	325 (1.9%)	562 (1.6%)
Black	142 (0.8%)	125 (0.7%)	267 (0.8%)
Others	239 (1.3%)	146 (0.8%)	385 (1.1%)
Unknown	49 (0.3%)	54 (0.3%)	103 (0.3%)
Drinking			
Current	17179 (94.4%)	16505 (96.0%)	33684 (95.2%)
Never	600 (3.3%)	312 (1.8%)	912 (2.6%)
Previous	406 (2.2%)	362 (2.1%)	768 (2.2%)
Unknown	15 (0.1%)	11 (0.1%)	26 (0.1%)
Smoking			
Current	975 (5.4%)	1306 (7.6%)	2281 (6.4%)
Never	11569 (63.6%)	9645 (56.1%)	21214 (59.9%)
Previous	5611 (30.8%)	6194 (36.0%)	11805 (33.4%)
Unknown	45 (0.2%)	45 (0.3%)	90 (0.3%)
Education			
Lower than secondary	1114 (6.1%)	1126 (6.6%)	2240 (6.3%)
Secondary	2848 (15.6%)	1764 (10.3%)	4612 (13.0%)
Vocational	1168 (6.4%)	916 (5.3%)	2084 (5.9%)
Higher education	4766 (26.2%)	4878 (28.4%)	9644 (27.3%)
Unknown	7964 (43.8%)	8180 (47.6%)	16144 (45.6%)

Summary statistics shown are mean (standard deviation) or *n* (%). Townsend Deprivation Index is a postcode-based measure of socioeconomic status, with smaller value indicating a lower level of deprivation. Weight was measured with BIA analyser. Fat mass and fat free mass were measured with dual-energy X-ray absorptiometry.

Web Table 2: Age-adjusted correlation between conventional body mass index and height, fat mass, fat free mass, waist circumference and fat mass percentage

	Females				Males			
	Overall	<50 Years	50-60 Years	>60 Years	Overall	<50 Years	50-60 Years	>60 Years
No.	267377	63139	92193	112045	222496	51776	71131	99589
Height	-0.13 (-0.13, -0.13)	-0.11 (-0.11, -0.10)	-0.12 (-0.13, -0.12)	-0.13 (-0.14, -0.13)	-0.06 (-0.07, -0.06)	-0.05 (-0.06, -0.04)	-0.06 (-0.06, -0.05)	-0.07 (-0.07, -0.06)
BF%	0.85 (0.85, 0.85)	0.87 (0.86, 0.87)	0.86 (0.85, 0.86)	0.84 (0.84, 0.84)	0.80 (0.80, 0.80)	0.81 (0.81, 0.82)	0.82 (0.81, 0.82)	0.79 (0.79, 0.80)
FM	0.94 (0.94, 0.94)	0.95 (0.95, 0.95)	0.94 (0.94, 0.94)	0.93 (0.93, 0.93)	0.92 (0.92, 0.92)	0.92 (0.92, 0.92)	0.92 (0.92, 0.93)	0.91 (0.91, 0.91)
FFM	0.68 (0.68, 0.68)	0.71 (0.71, 0.72)	0.71 (0.70, 0.71)	0.68 (0.68, 0.68)	0.64 (0.64, 0.64)	0.67 (0.67, 0.67)	0.67 (0.67, 0.67)	0.63 (0.63, 0.64)
Weight	0.92 (0.92, 0.92)	0.93 (0.93, 0.93)	0.92 (0.92, 0.92)	0.91 (0.91, 0.91)	0.88 (0.88, 0.88)	0.89 (0.88, 0.89)	0.89 (0.89, 0.89)	0.88 (0.88, 0.88)
WC	0.88 (0.88, 0.88)	0.89 (0.89, 0.89)	0.88 (0.88, 0.88)	0.87 (0.86, 0.87)	0.88 (0.88, 0.88)	0.88 (0.88, 0.88)	0.88 (0.88, 0.89)	0.87 (0.87, 0.87)
WHR	0.46 (0.45, 0.46)	0.47 (0.47, 0.48)	0.47 (0.46, 0.47)	0.43 (0.43, 0.44)	0.59 (0.59, 0.59)	0.62 (0.61, 0.62)	0.60 (0.60, 0.61)	0.58 (0.58, 0.59)
WHtR	0.89 (0.88, 0.89)	0.90 (0.89, 0.90)	0.89 (0.89, 0.89)	0.88 (0.88, 0.88)	0.88 (0.88, 0.88)	0.89 (0.89, 0.89)	0.90 (0.90, 0.90)	0.89 (0.89, 0.89)

FM: fat mass. FFM: fat free mass. BF%: body fat percentage. WC: waist circumference. WHR: waist-to-hip ratio. WHtR: waist-to-height ratio.

Web Table 3: Age-adjusted correlation coefficients of old and new BMI with height and fatness measures in females and males in training and testing data sets

	Training Set		Testing Set—Main		Testing Set—DXA	
	BMI_old	BMI_new	BMI_old	BMI_new	BMI_old	BMI_new
Females						
Power value	2.00	1.39	2.00	1.39	2.00	1.39
Height	-0.12	0.00	-0.12	0.00	-0.12	0.01
Fat percentage	0.85	0.86	0.85	0.86	0.83	0.82
Weight	0.92	0.96	0.92	0.96	0.92	0.96
Fat mass	0.94	0.96	0.94	0.96	0.94	0.96
Fat free mass	0.70	0.77	0.70	0.76	0.57	0.64
WC	0.88	0.89	0.88	0.89	0.86	0.88
WHR	0.45	0.45	0.45	0.45	0.43	0.42
WHtR	0.89	0.87	0.89	0.87	0.88	0.86
Males						
Power value	2.00	1.77	2.00	1.77	2.00	1.77
height	-0.06	0.00	-0.06	-0.00	-0.06	-0.00
Fat percentage	0.81	0.81	0.81	0.80	0.77	0.77
Weight	0.88	0.91	0.88	0.91	0.88	0.91
Fat mass	0.92	0.93	0.92	0.93	0.90	0.91
Fat free mass	0.66	0.69	0.65	0.69	0.56	0.60
WC	0.88	0.89	0.88	0.89	0.87	0.88
WHR	0.60	0.60	0.60	0.60	0.59	0.59
WHtR	0.89	0.88	0.89	0.88	0.89	0.88

(Continued)

	Testing Set: White		Testing Set: Asian		Testing Set: Black		Testing Set: Other	
	BMI_old	BMI_new	BMI_old	BMI_new	BMI_old	BMI_new	BMI_old	BMI_new
Females								
Power value	2.00	1.39	2.00	1.39	2.00	1.39	2.00	1.39
Height	-0.13	-0.00	-0.10	0.03	-0.11	0.01	-0.05	0.07
Fat percentage	0.85	0.86	0.85	0.87	0.83	0.84	0.85	0.86
Weight	0.92	0.96	0.91	0.96	0.92	0.96	0.92	0.96
Fat mass	0.94	0.96	0.93	0.96	0.93	0.96	0.94	0.97
Fat free mass	0.70	0.76	0.69	0.76	0.74	0.80	0.72	0.78
WC	0.88	0.89	0.84	0.85	0.87	0.88	0.87	0.88
WHR	0.46	0.45	0.37	0.36	0.35	0.34	0.39	0.38
WHtR	0.89	0.87	0.85	0.83	0.88	0.85	0.88	0.85
Males								
Power value	2.00	1.77	2.00	1.77	2.00	1.77	2.00	1.77
height	-0.07	-0.01	-0.00	0.06	0.00	0.06	-0.02	0.04
Fat percentage	0.81	0.81	0.77	0.77	0.75	0.75	0.78	0.78
Weight	0.88	0.91	0.87	0.90	0.90	0.92	0.89	0.91
Fat mass	0.92	0.93	0.89	0.91	0.90	0.91	0.92	0.92
Fat free mass	0.65	0.69	0.67	0.71	0.71	0.74	0.68	0.71
WC	0.88	0.89	0.86	0.87	0.88	0.88	0.87	0.88
WHR	0.60	0.60	0.55	0.55	0.55	0.55	0.55	0.54
WHtR	0.89	0.89	0.86	0.85	0.88	0.87	0.88	0.87

Old BMI used the power value of 2.00 in the formula. New BMI was derived based on the criteria of height-independence, with the power value of height to minimize the correlation between the index and body fat percentage. BMI: body mass index. Ethnicity-specific testing sets were subsets of the main testing set, in which body composition was measured with BIA. In testing set-DXA, body composition was measured with DXA. BF%: body fat percentage. WC: waist circumference. WHR: waist-to-hip ratio. WHtR: waist-to-height ratio.

Web Figure 2: Box plot of conventional and female-specific BMI according to weight groups defined by conventional BMI in females

Females

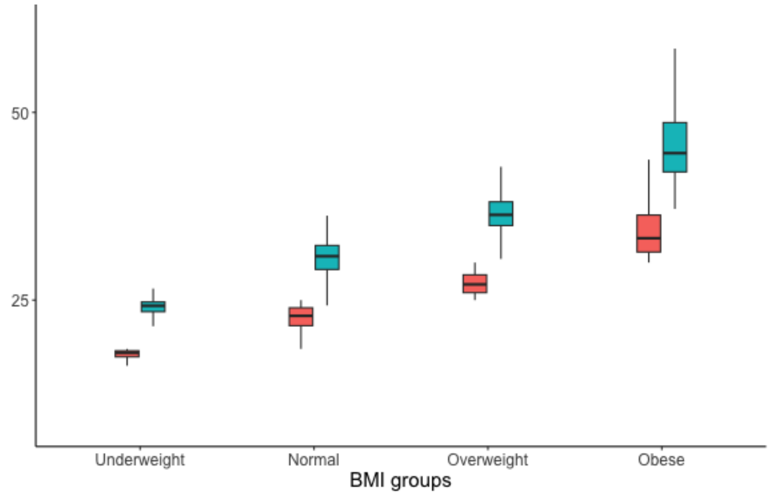

BMI_old [mean (SD)]	17.7 (0.75)	22.7 (1.56)	27.2 (1.41)	34.5 (4.14)
BMI_new [mean (SD)]	24.0 (1.12)	30.6 (2.17)	36.5 (2.04)	46.1 (5.59)

Males

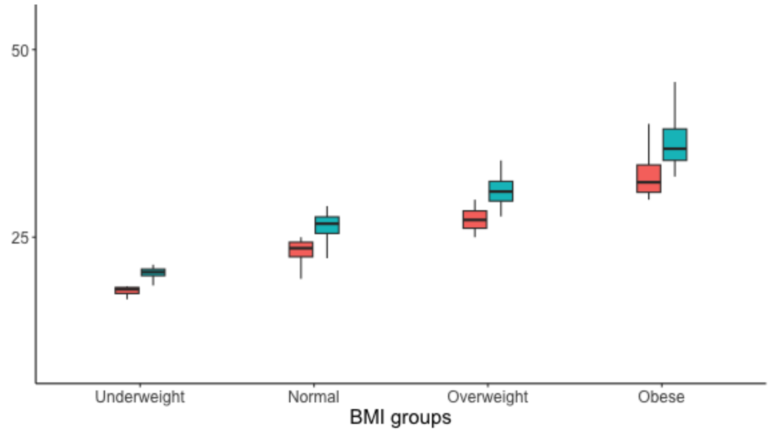

BMI_old [mean (SD)]	17.9 (0.51)	23.2 (1.38)	27.4 (1.39)	33.3 (3.33)
BMI_new [mean (SD)]	20.3 (0.66)	26.4 (1.58)	31.1 (1.59)	37.9 (3.79)

Green: Female-specific BMI. Red: conventional BMI. Definition for BMI groups ($kg/m^{2.00}$): underweight BMI_old < 18.5, normal 18.5 < BMI_old < 25, overweight 25 < BMI_old < 30, obese BMI_old > 30. BMI_old: conventional BMI, weight/height^2. BMI_new: weight/height $^{1.39}$ for females and weight/height $^{1.77}$ for males. The boxplot shows 25th percentile – 1.5*interquartile, 25th percentile, median, 75th percentile, 1.5*interquartile range + 75th percentile. Interquartile = 75th percentile − 25th percentile.

Web Table 4: Association between all-cause death with old and new BMI in females and males in the testing set

	Old BMI		New BMI	
	Events/Total	HR (95%CI)	Events/Total	HR (95%CI)
Females				
Fifth_1	586 / 10701	Reference	623 / 10697	Reference
Fifth_2	581 / 10691	0.88 (0.78, 0.98)	559 / 10694	0.83 (0.74, 0.93)
Fifth_3	657 / 10698	0.91 (0.82, 1.02)	667 / 10697	0.91 (0.81, 1.01)
Fifth_4	732 / 10693	0.95 (0.85, 1.06)	721 / 10693	0.93 (0.84, 1.04)
Fifth_5	974 / 10693	1.32 (1.19, 1.47)	960 / 10695	1.31 (1.18, 1.45)
AIC	73871.57		73862.40	
Males				
Fifth_1	938 / 8906	Reference	952 / 8902	Reference
Fifth_2	908 / 8897	0.92 (0.84, 1.01)	918 / 8900	0.94 (0.85, 1.02)
Fifth_3	907 / 8898	0.94 (0.85, 1.03)	899 / 8899	0.93 (0.85, 1.02)
Fifth_4	991 / 8903	0.96 (0.87, 1.05)	986 / 8905	0.95 (0.87, 1.04)
Fifth_5	1322 / 8896	1.30 (1.20, 1.42)	1311 / 8894	1.31 (1.20, 1.43)
AIC	103577.36		103586.14	

Model was adjusted for smoking, drinking, deprivation index, age, and region.
AIC: Akaike information criterion. BMI: body mass index. CI: confidence interval. HR: hazard ratio.
